# Genetic screening for hypertension and COVID-19 reveals functional variation of *SPEG* potentially associated with severe COVID-19 in women

**DOI:** 10.3389/fgene.2022.1041470

**Published:** 2023-01-04

**Authors:** Yu-Si Luo, Xiang-Chun Shen, Wei Li, Guo-Feng Wu, Xiao-Meng Yang, Ming-Yang Guo, Fang Chen, Hu-Yan Shen, Ping-Ping Zhang, Han Gao, Ying Nie, Jia-Hong Wu, Rong Mou, Ke Zhang, Zhong-Shan Cheng

**Affiliations:** ^1^ Department of Emergency, The Affiliated Hospital of Guizhou Medical University, Guiyang, China; ^2^ The Key and Characteristic Laboratory of Modern Pathogenicity Biology, School of Basic Medical Sciences, Guizhou Medical University, Guiyang, China; ^3^ The High Efficacy Application of Natural Medicinal Resources Engineering Center of Guizhou Province, School of Pharmaceutical Sciences, Guizhou Medical University, Guiyang, China; ^4^ State Key Laboratory for Functions and Applications of Medicinal Plants, Guizhou Medical University, Guiyang, China; ^5^ Department of Cardiovascular, The Affiliated Hospital of Guizhou Medical University, Guiyang, China; ^6^ Center for Applied Bioinformatics, St. Jude Children’s Research Hospital, Memphis, TN, United States

**Keywords:** hypertension, severe COVID-19, GWAS, *SPEG*, women, cardiomyocyte

## Abstract

The coronavirus disease 2019 (COVID-19) pandemic, caused by the severe acute respiratory syndrome coronavirus 2 (SARS-CoV-2), has led to more than 6.4 million deaths worldwide. The prevalent comorbidity between hypertension and severe COVID-19 suggests common genetic factors may affect the outcome of both diseases. As both hypertension and severe COVID-19 demonstrate sex-biased prevalence, common genetic factors between the two diseases may display sex-biased differential associations. By evaluating COVID-19 association signals of 172-candidate hypertension single nucleotide polymorphisms (SNPs) derived from more than 1 million European individuals in two sex-stratified severe COVID-19 genome-wide association studies from UK BioBank with European ancestry, we revealed one functional cis expression quantitative trait locus of *SPEG* (rs12474050) showing sex-biased association with severe COVID-19 in women. The risk allele rs12474050*T associates with higher blood pressure. In our study, we found it is significantly correlated with lower *SPEG* expression in muscle-skeletal but with higher expression in both brain cerebellum and cerebellar hemisphere. Additionally, nominal significances were detected for the association between rs12474050*T and lower *SPEG* expression in both heart left ventricle and atrial appendage; among these tissues, the *SPEG* expression is nominally significantly higher in females than in males. Further analysis revealed *SPEG* is mainly expressed in cardiomyocytes in heart and is upregulated upon SARS-CoV-2 infection, with significantly higher upregulation of *SPEG* only observed in female but not in male COVID-19 patients compared to both normal female and male individuals, suggesting upregulation of *SPEG* is a female-specific protective mechanism against COVID-19 induced heart damage. Taken together, our analyses suggest the involvement of *SPEG* in both hypertension and severe COVID-19 in women, which provides new insights for sex-biased effect of severe COVID-19 in women.

## Introduction

The coronavirus disease 2019 (COVID-19) pandemic, caused by the severe acute respiratory syndrome coronavirus 2 (SARS-CoV-2), has been quickly spreading in more than 200 countries and overwhelmingly challenging the global population. Since November 2019, there are 591,683,619 cases have been reported and 6,443,306 deaths have been documented (WHO, 2022). The overall mortality rate of COVID-19 is approximately 1.09% at the time of writing.

Numerous studies, including observational and retrospective research, had demonstrated that hypertension was the most frequent comorbidity of COVID-19 patients and was reported as a common and independent risk factor for the severity and mortality of patients suffered from COVID-19 (Chen et al., 2021; Dai et al., 2022). Typically, hypertension is measured based on diastolic blood pressure (DBP) and systolic blood pressure (SBP), and patients with DBP ≥ 90 mmHg or SBP ≥ 140 mmHg are diagnosed with hypertension (Chen et al., 2021). A recent study suggested hypertension contributed 2.5-fold increased risk of disease severity and mortality in COVID-19 patients (Lippi et al., 2020). Nevertheless, SARS-CoV-2 infection is also able to induce hypertension post COVID-19, named as Post-COVID-19 hypertension and highlighted by two studies from Turkey research groups (Akpek, 2022; Uysal et al., 2022). According to the data from the retrospective cohort study presented by Akpek et al., the DBP and SBP of 153 eligible COVID-19 patients (mean age 46.5 ± 12.7 years) were statistically higher in the post COVID-19 period than on admission (Akpek, 2022). The other prospective multicenter study reported the DBP and SBP of 100 eligible COVID-19 young patients (mean age 189.45 ± 23.78 months) were remarkably higher in the post COVID-19 period than on admission (Uysal et al., 2022). The potential cause of Post-COVID-19 hypertension by SARS-CoV-2 infection may be due to the shift of renin-angiotensin system (RAS) related balance from Mas [angiotensin converting enzyme 2 (ACE2)/angiotensin (Ang) 1-7/Mas] axis to RAS [Ang converting enzyme (ACE)/Ang II/Ang II type I receptor (AT1R)] axis (Uysal et al., 2022) upon SARS-CoV-2 infection. A new study further revealed that severe COVID-19 induced autoantibodies against Ang II is correlated with blood pressure dysregulation and COVID-19 severity (Briquez et al., 2022). Additionally, infection of SARS-CoV-2 might also lead to aggravation of pre-existing hypertension and damages of other organs, such as heart, which consequently contributed to more severe clinical consequences (Wang et al., 2020).

In order to explore potentially sex-biased genetic factors influencing both COVID-19 severity and hypertension, we performed an integrative screening of known hypertension association single nucleotide polymorphisms (SNPs) that are also cis-expression quantitative trait loci (cis-eQTLs) in severe COVID-19 GWASs stratified by sex. Our analyses reveal that only one cis-eQTL, rs12474050, mapped to the gene striated preferentially expressed protein kinase (*SPEG*) encoding the protein SPEG, shown significant, sex-biased association with severe COVID-19 in women. Further scientific literature reviewing underpinned that *SPEG* played critical roles in the development, maintenance, and function of cardiac and skeletal muscles (Campbell et al., 2021; Luo et al., 2021), and the expression of *SPEG* is suggestively higher in muscle skeletal, heart atrial appendage, and heart left ventricle in females than in males. We thus investigated the potential link between sex-biased expression of *SPEG* and COVID-19 severity in females.

## Materials and methods

### Selection of hypertension SNPs that are cis-eQTLs in GTEx

According to Evangelou et al. (Evangelou et al., 2018), hypertension association SNPs (*n* = 172 lead SNPs with independent genome-wide significant association signals) that were also cis-eQTLs in GTEx database (GTEx, 2020) were obtained. These SNPs were derived from a hypertension GWAS conducted over 1 million European individuals. Two hypertension traits, including DBP and SBP were studied by the hypertension GWAS. Among these 172 cis-eQTLs, the top five tissues harboring the largest number of these cis-eQTLs include adipose subcutaneous (*n* = 43), artery tibial (*n* = 41), artery aorta (*n* = 39), nerve tibial (n = 39), muscle skeletal (*n* = 38). We referred to these 172-candidate hypertension association SNPs as hypertension cis-eQTLs.

### Evaluation of hypertension cis-eQTLs in severe COVID-19 GWASs in European populations

To screen potential functional SNPs associated with both hypertension and severe COVID-19, we evaluated the association signals of these candidate hypertension cis-eQTLs that were reported as independent genome-wide significant SNPs in the hypertension GWAS in the current severe COVID-19 GWASs of European ancestry that were performed separately by sex, the summary statistics of which were freely available from GRASP database (Thibord et al., 2022). Details for the two GWASs are as follows: the first GWAS, severe COVID-19 positive vs. non-severe COVID-19 positive in European (EUR) Female UK BioBank (UKBB) tested positive samples, has 283 cases and 7,113 controls; the second GWAS, severe COVID-19 positive vs. non-severe COVID-19 positive in EUR Male UKBB tested positive samples, has 565 cases and 6,093 controls. The rationale to focus on severe COVID-19 by sex is due to the observation that sex is a risk factor for both hypertension and severe COVID-19. Furthermore, due to the nature that these 172 cis-eQTLs associated with hypertension were derived from European samples, we therefore only focused on severe COVID-19 GWASs conducted on European samples.

We conducted sex-biased differential association analysis for these 172 hypertension cis-eQTLs extracted from the sex-stratified severe COVID-19 GWASs. Sex-biased differential association analysis was performed with Z-test according to Thibord et al. (Thibord et al., 2022). See the below formula for calculating delta Z-score (ΔZ-score) and its corresponding *p*-value. Bonferroni correction was used to adjust the significance of differential association between sex, with the threshold set at *p* < 0.05/172 = 3e-4. Only one cis-eQTL, rs12474050, mapped to *SPEG*, passed the statistical significance threshold, with its ΔZ-score equal to 3.75 and differential Z-score *p*-value equal to 1.8e-4. rs12474050 was also a top SNP showing suggestive association with severe COVID-19 in females (*p* = 1.78e-4; beta = 0.36; se = 0.10). In the corresponding severe COVID-19 GWAS of males, the cis-eQTL was not significant (*p* = 0.23; beta = −0.08; se = 0.07).

$$\Delta Z-score=\frac{female.snp.\beta -male.snp.\beta }{\sqrt{\left(female.snp.se^{2}+male.snp.se^{2}\right)}}$$

$$p=pnorm\left(-\left|\Delta Z-score\right|\right)*2$$

### Functional annotation for *SPEG* cis-eQTL rs12474050

As rs12474050 was reported as a cis-eQTL of *SPEG* in GTEx database V7 by the hypertension GWAS, we further evaluated the association between rs12474050 and *SPEG* expression in GTEx database V8 (GTEx, 2020). In addition, we also investigated *SPEG* expression among 49 GTEx tissues by sex and inferred tissue specific biological functions of *SPEG* by determining whether *SPEG* was highly expressed in a specific tissue.

### Expression analysis of *SPEG* in multiple GTEx tissues

Similar to our previous analysis (Luo et al., 2022), bulk RNAseq transcript per million (TPM) matrix data of 49 tissues, as well as sex information of these samples from GTEx database V8 (GTEx, 2020), were downloaded from GTEx Portal. By matching samples with sex information, as well as tissue information, we determined differential expression of *SPEG* between males and females across multiple GTEx tissues with ANOVA test implemented in the procedure proc GLM using SAS OnDemand for Academics.

We also evaluated *SPEG* expression among different single cells from eight tissues, including breast mammary tissue, esophagus mucosa, esophagus muscularis, heart left ventricle, lung, muscle skeletal, prostate, skin sun exposed lower leg, using the visualization tool ‘GTEx Multi-Gene Single Cell Query’ from GTEx Portal.

### Evaluation of *SPEG* expression upon SARS-CoV-2 infection

To determine the expression of *SPEG* upon SARS-CoV-2 infection, we firstly re-analyzed publicly available single cell expression data that were related to COVID-19 and stored in the UCSC Cell Browser (Speir et al., 2021). By querying the keyword “COVID” for the option of disease type, we obtained 13 COVID-19 related single cell data sets from UCSC Cell Browser (accessed on 8 August 2022; see Supplementary Material for these 13 COVID-19 single cell data sets). After evaluating *SPEG* expression across the above 13 COVID-19 single cell expression data sets in UCSC Cell Browser, we revealed that *SPEG* is highly expressed in cardiomyocyte cells in heart tissue based on single cell data from the dataset “Cellular Targets of SARS-CoV-2” (Melms et al., 2021). Thus, we selected the heart single cell data set “Cellular Targets of SARS-CoV-2” for deep investigation of *SPEG* expression. The expression data matrix for the heart tissue single cell data set, as well as its sample meta data and Uniform Manifold Approximation and Projection (UMAP) for all single cells, are provided by UCSC Cell Browser.

Since our aim is to evaluate whether *SPEG* expression shows sex-biased differential expression between females and males for both COVID-19 patients and healthy controls, we first performed sample level differential expression analysis for the data set according to Trump et al. (Trump et al., 2021). We linked the normalized read count matrix and clinical information of these COVID-19 samples, such as sex and COVID-19 status, as well as single cell UMAP coordinates, for further analyses using SAS OnDemand for Academics. Differential expression of *SPEG* between healthy controls and COVID-19 patients by sex were conducted for each single cell type at sample level. In total, there were four groups, including COVID-19 females (*n* = 6), COVID-19 males (*n* = 13), healthy females (*n* = 12), and healthy males (*n* = 16), subjected to analysis. Samples without specific single cell types were not included in the final differential expression or other comparisons, such as comparisons of cell frequency and cell percentage. We used median log2 (expression of *SPEG* + 1) in each sample for different single cell types and subsequently performed differential gene expression analysis for *SPEG*. We applied the option lsmeans from SAS proc GLM procedure to perform multiple comparisons and the *p* values for multiple comparisons among different groups in each single cell type were adjusted with Tukey adjustment. The significance threshold was set at adjusted *p* ≤ 0.05. In terms of cell counts and cell percentage for each single cell type from each sample, as well as cell counts and cell percentage of *SPEG* expressed cells in each sample, we compared these parameters across the above four groups using the same SAS procedure and used the same adjusted *p*-value threshold to determine statistical significance.

Alternatively, we also conducted single cell level differential expression analysis for *SPEG* for each single cell type by aggregating all cells of specific single cell type together among the four patient groups, including COVID-19 females, COVID-19 males, healthy females, and healthy males. This analysis was performed using R package Seurat (Hao et al., 2021) (see details of analysis procedures in Supplementary Material). Briefly, normalization was performed to obtain relative gene expression abundances between cells by scaling count data with default setting in Seurat. Differential gene expression analysis of *SPEG* was conducted among each cell cluster defined by (Melms et al., 2021) using the default Wilcoxon Rank Sum test in Seurat. As only two cell types display *SPEG* expression, including cardiomyocyte and vascular smooth muscle cell (VSMC), differential expression analysis for *SPEG* was only conducted among the two cell types, with multiple adjusted *p*-value threshold set at *p* < 0.05/(2 × 6) = 4e-3 to determine significance. Additionally, the average log2 (fold change) of *SPEG* expression and the percent of cells expressing *SPEG* between two compared patient groups were generated by Seurat.

We also evaluated *SPEG* expression in cardiomyocytes upon SARS-CoV-2 infection in a bulk RNAseq dataset downloaded from NCBI gene expression omnibus (GEO) (accession number: GSE156754) (Perez-Bermejo et al., 2021). This published data set evaluated genes responding to SARS-CoV-2 infection among multiple cardiac cells, including human induced pluripotent stem cell-derived cardiomyocytes, cardiac fibroblasts, and endothelial cells. We obtained the normalized read counts of *SPEG* and compared *SPEG* expression between SARS-CoV-2 infected cardiac cells and its corresponding mock controls, differential gene expression analyses were performed using Student’s t-test with the software Prism GraphPad. Multiple adjustment *p*-value threshold was set at *p* ≤ 0.008 (0.05/6).

## Results

**FIGURE 1:**
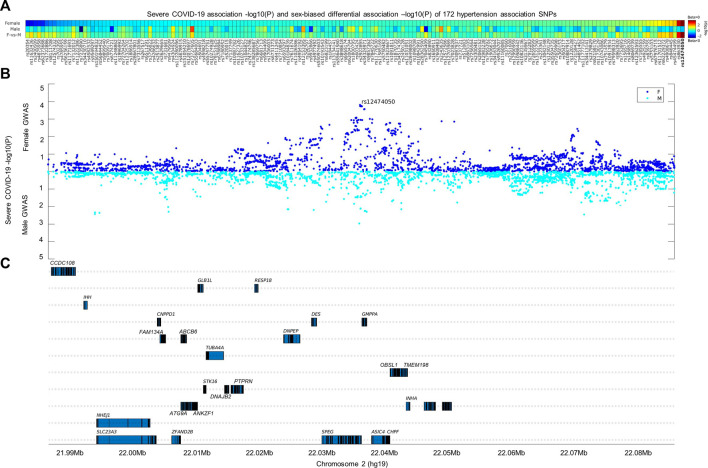
Integrative analysis of 172-hypertension association SNPs with severe COVID-19 genome-wide association signals by sex. The heatmap in the **(A,B)** display the COVID-19 association signals of these 172-hypertension association SNPs separated by sex, as well as sex-biased differential associations signals at the **(C)** of the heatmap (see method for calculation of sex-biased differential association *p* values); these SNPs are genome-wide significantly associated with hypertension (p < 5e-8) and are also cis expression quantitative trait loci (cis-eQTLs) according to Evangelou et al. (Evangelou et al., 2018). Only one SNP, rs12474050 (highlighted in red on the end-right side of the heatmap), mapped to *SPEG*, demonstrates sex-biased association (differential association *p*-value = 1.8e-4; passed the multiple adjusted *p*-value threshold of p < 3e-4) with severe COVID-19 in females. The SNP demonstrates suggestive association with severe COVID-19 females (*p* = 1.8e-4) and but not with severe COVID-19 males (*p* = 0.23). The scatterplots in the middle panel illustrate the severe COVID-19 association signals by sex for common SNPs (minor allele frequency >0.05) within a window of 1-Mbp where rs12474050 is located at the center, and genes covered in the window are located in the lower panel. Note: the COVID-19 GWAS association signals for females and males are colored in green and blue, respectively; the sex-biased multiple adjusted association *p*-value was set as *p* ≤ 3e-4, i.e., 0.05/172.

The frequent observation of comorbidity between hypertension and severe COVID-19 promoted us to search for genetic factors associated with both diseases. Based on a previously published large-scale hypertension GWAS conducted over 1 million European individuals (Evangelou et al., 2018), we selected 172 independent hypertension association SNPs that were also cis-eQTLs for evaluation in two severe COVID-19 GWASs conducted separately by sex from UK BioBank with European ancestry. In the sex-biased differential association analysis, only one SNP out of 172-candidate SNPs passed the multiple adjusted association *p*-value threshold (*p* < 3e-4). The SNP rs12474050 (sex-biased differential association *p* = 1.8e-4) is a cis-eQTL of *SPEG* and emerged with suggestive association with severe COVID-19 in women (*p* = 1.8e-4; beta = 0.36; se = 0.10) but not in men (*p* = 0.23; beta = –0.08; se = 0.07) (Figure 1). We thus evaluated local COVID-19 association signals in a 1-Mbp window where rs12474050 located at the center in the two severe COVID-19 GWASs by sex. The local Manhattan plot by sex in Figure 1 demonstrated that rs12474050 was one of the top COVID-19 association signals in the female COVID-19 GWAS, with more SNPs showing suggestive associations (p < 1e-3) in females than in males, suggesting rs12474050 is a sex-biased genetic factor showing association with COVID-19 in females. Collectively, our integrative genetic screening of both hypertension and severe COVID-19 among European samples revealed rs12474050 is a sex-biased marker associated with severe COVID-19 in women.

**FIGURE 2:**
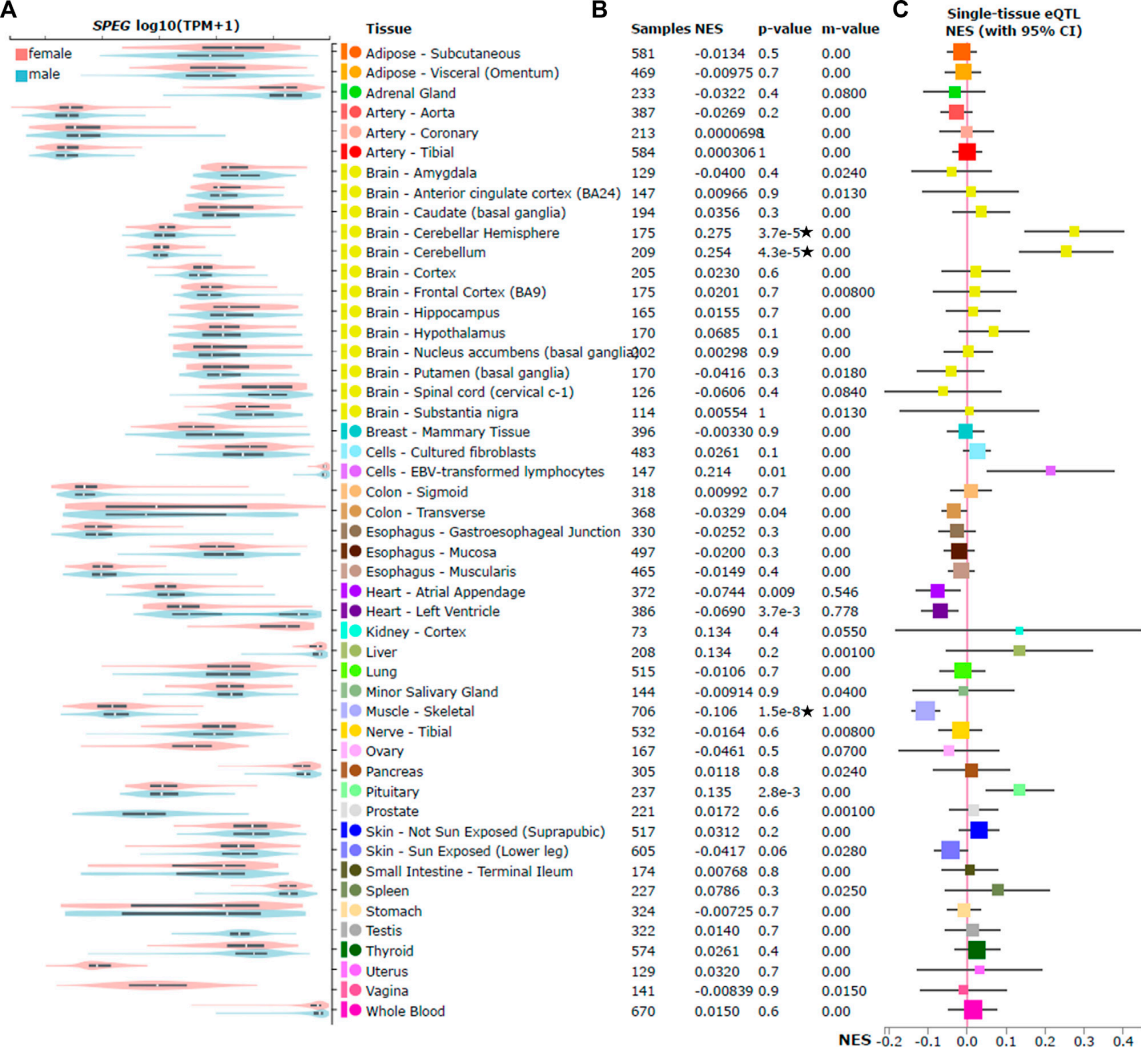
*SPEG* expression and cis expression quantitative trait locus (cis-eQTL) analysis of rs12474050 across 49 GTEx tissues. Violin plots on the **(A)** illustrates the expression of *SPEG* among 49 GTEx tissues by sex; among these tissues, only breast mammary tissue show statistically (nominal *p* = 7.1e-7) higher expression of *SPEG* in females, although in heart left ventricle the SNP is nominally associated with *SPEG* expression (nominal *p* = 0.004) but not passed the strict multiple adjustment *p*-value threshold *p* ≤ 0.001 (0.05/49). Meanwhile, no significant differences between females and males for *SPEG* expression in the two brain tissues (brain cerebellar hemisphere and brain cerebellum that display significant eQTL signals) where rs12474050 is positively correlated with *SPEG* expression. **(B)** displays the multi-tissue cis-eQTL plot for rs12474050, with the COVID-19 risk allele rs12474050*T mostly, but negatively associated with *SPEG* expression in muscle skeletal that passes the multiple adjusted *p*-value threshold of *p* ≤ 0.001. Note: star labeled along with the nominal cis-eQTL *p*-value indicates that the *p*-value passes the multiple adjustment *p*-value threshold. Nevertheless, the risk allele is positively associated with *SPEG* expression in two brain tissues, including cerebellar hemisphere and cerebellum. Details of cis-eQTL results are also provided, including tissue name, sample size, normalized effect size (NES), the posterior probability of rs12474050 for each tissue in cis-eQTL meta-analysis (m-value), and unadjusted cis-eQTL association *p*-value. **(C)** is the NES forest plot of rs12474050, with the mean of NES represented by square that are weighted and colored according to size of NES and tissue type, respectively.

As rs12474050 was reported as a cis-eQTL of *SPEG* by the previously published hypertension GWAS (Evangelou et al., 2018), in which the GTEx database V7 was used to annotate these cis-eQTLs, we further evaluated its association with *SPEG* expression across 49 tissues in GTEx database V8 (GTEx, 2020). We confirmed that rs12474050 is indeed a cis-eQTL of *SPEG* (Figure 2) and the COVID-19 risk allele rs12474050*T negatively associates with *SPEG* expression among muscle skeletal (nominal *p* = 1.5e-8; beta = −0.106), heart atrial appendage (nominal p = 9e-3; beta = −0.074), and heart left ventricle (nominal *p* = 3.7e-3; beta = −0.069), although only in the first tissue the SNP passed the multiple adjusted *p*-value threshold of *p* ≤ 0.001 (0.05/49). In addition, rs12474050*T demonstrates positive association with *SPEG* expression in two brain tissues, including brain cerebellar hemisphere (nominal *p* = 3.7e-5; beta = 0.28) and brain cerebellum (nominal *p* = 4.3e-5; beta = 0.25), the association *p* values in which all passed the multiple adjusted *p*-value threshold. Nevertheless, in the cis-eQTL meta-analysis, the posterior probability (m-value in Figure 2) of rs12474050 in each tissue revealed that muscle skeletal, heart atrial appendage, and heart left ventricle all display m-value >0.5, indicating the association pattern of rs12474050 with *SPEG* is more constant between these three tissues along with the majority of other tissues. In short, the risk allele rs12474050*T is a cis-eQTL of *SPEG* and associated with lower *SPEG* expression among the majority of GTEx tissues, particularly with significant association in muscle skeletal and suggestive associations in heart atrial appendage and heart left ventricle.

Since rs12474050*T was only found suggestively associated with severe COVID-19 in females but not in males, we questioned whether *SPEG* was differentially expressed between sex in muscle skeletal, heart atrial appendage, and heart left ventricle, as well as breast mammary tissue. We revealed that among the first three tissues *SPEG* expression was indeed suggestively higher in females than in males (Figure 2; nominally differential expression *p* values: 0.02, 0.03, and 0.004, respectively). Additionally, we also evaluated *SPEG* expression in the breast mammary tissue, which is biologically different between females and males, it revealed that females demonstrated statistically higher expression of *SPEG* (nominal *p* = 7.1e-7). Nevertheless, when tested the eQTL association in two brain tissues, including brain cerebellar hemisphere and brain cerebellum, there were no significant differences between females and males for *SPEG* expression. In conclusion, apart from the genetic effect of rs12474050, sex is another factor affecting *SPEG* expression in breast mammary tissue, muscle skeletal, heart atrial appendage, and heart left ventricle.

**FIGURE 3:**
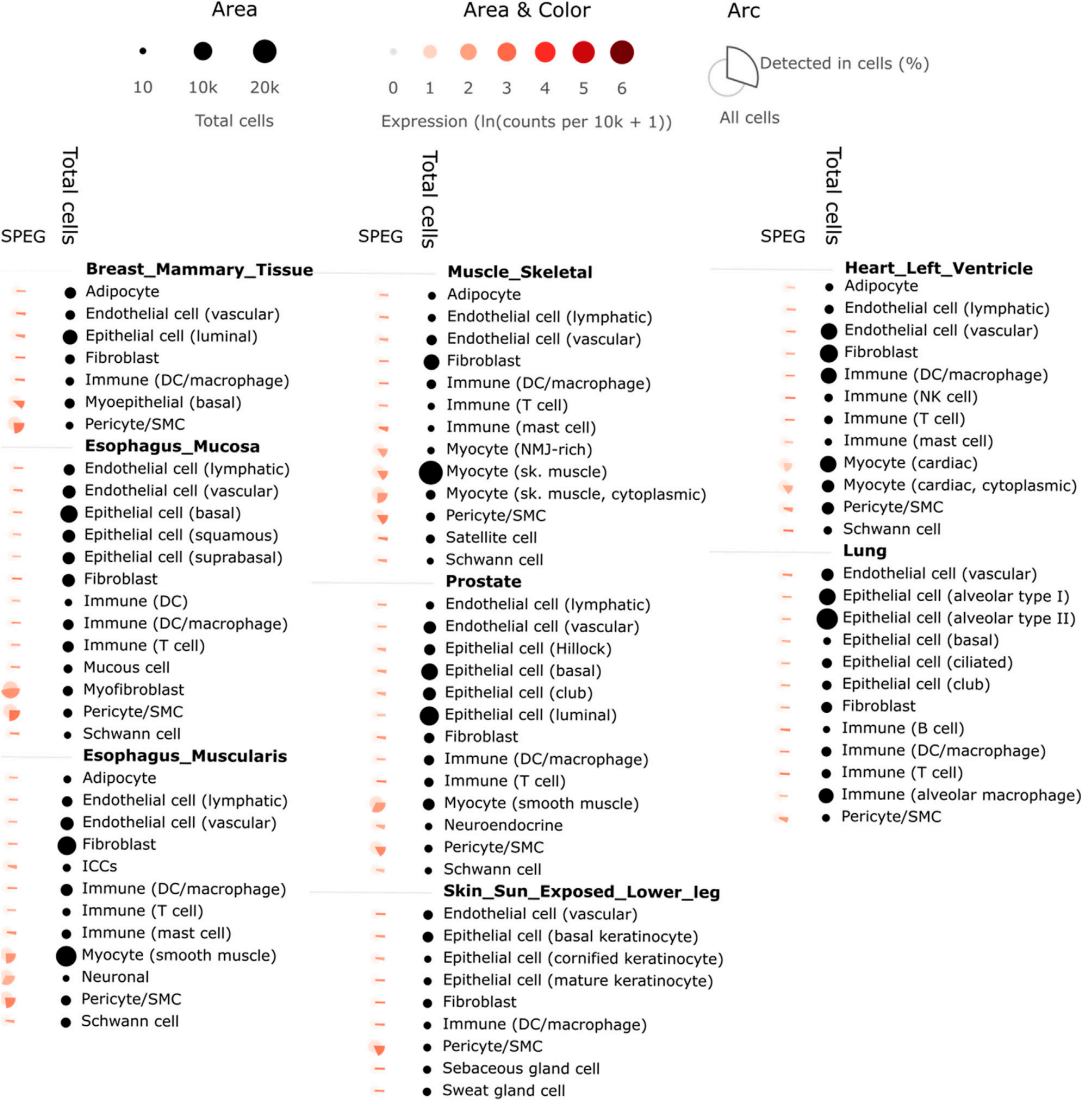
Single cell analysis of *SPEG* expression among 8 GTEx tissues reveals *SPEG* expression mainly detected in myocyte related single cell types. Different single cell types from 8 tissues, including breast mammary tissue, esophagus mucosa, esophagus muscularis, heart left ventricle, lung, muscle skeletal, prostate, skin sun exposed lower leg, are determined for the % of cells expression *SPEG*. Higher % of cells expressing *SPEG* are found among myocyte related cell types. Low level of *SPEG* expression is detected among immune cells and epithelial cells.

**FIGURE 4:**
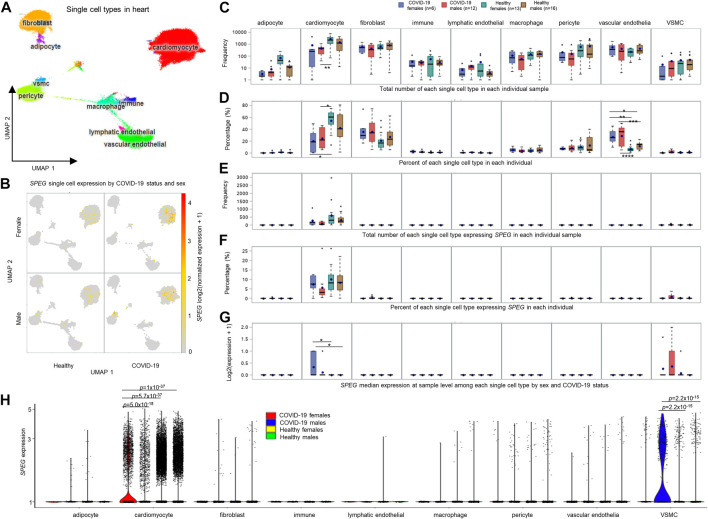
Single cell analysis of heart tissue reveals that *SPEG* expression is up-regulated in cardiomyocytes upon SARS-CoV-2 infection. **(A)** Uniform Manifold Approximation and Projection (UMAP) analysis of heart single cell data set derived from healthy controls (13 females and 16 males) and COVID-19 patients (6 females and 12 males) published by Melms et al. (Melms et al., 2021). **(B)** UMAPs divided by COVID-19 status and sex illustrating the cardiomyocytes is the major cell type in heart to express *SPEG*. **(C)** Total number of each single cell type in each individual sample compared among different groups, including COVID-19 females, COVID-19 males, healthy females, and healthy males. **(D)** Percent of each single cell type in each individual measured and evaluated across different sample groups. **(E)** Total number of each single cell type expressing *SPEG* in each individual sample determined across different groups. **(F)** Percent of each single cell type expressing *SPEG* in each individual examined among different sample groups. **(G)** Differential gene expression analysis of *SPEG* median expression at sample level among each single cell type by sex and COVID-19 status. Note: For each single cell type, multiple comparison *p* values were subjected to Tukey adjustment using the lsmeans statement in SAS proc GLM procedure, with the adjusted statistical significance represented by * (*p* ≤ 0.05), ** (*p* ≤ 0.01), *** (*p* ≤ 0.001), and **** (*p* ≤ 0.0001). The box-and-whisker plots display the mean (dot within box), median (line inside the box), inter-quantile interval (box), minimum (lowest value of whisker) and maximum (maximum value of whisker), with outliers represented by dots up or down the whiskers. **(H)** Differential gene expression analysis of *SPEG* expression at single cell level by aggregating all cells of each single cell type by sex and COVID-19 status. Nominal adjusted *p* values were determined using R package Seurat (Hao et al., 2021). All these nominal *p* values pass the multiple adjustment *p*-value threshold of *p* < 0.05/(2 × 6) = 4e-3, as there are only 2 cell types, including cardiomyocyte and vascular smooth muscle cell (VSMC) displaying *SPEG* expression.

To investigate the potential involvement of *SPEG* in severe COVID-19, we determined *SPEG* expression among 13 single cell RNAseq data sets collected by UCSC Cell Browser and one standard along bulk RNAseq data related to cardiac cells upon SARS-CoV-2 infection from GEO (see method section for detail of these data sets). Most of these single cell data sets related to COVID-19 from UCSC Cell Browser are linked to epithelium cells and immune cells responding to SARS-CoV-2 infection. However, the expression of *SPEG* was rare among different epithelium and immune cells based on evaluation of these single cell data sets in UCSC Cell Browser (data not shown). This is in line with the observation of *SPEG* in GTEx single cell data is mainly expressed in myofibroblast and myocyte and <50% cells expression *SPEG* among all different single cell types (Figure 3); for cardiomyocytes from heart left ventricle, only 21% of myocytes (cardiac) and 16% of myocytes (cardiac, cytoplasmic) express *SPEG*. For single cell data from UCSC Cell Browser, only in heart tissue, *SPEG* was observed to be mainly expressed in the cardiac cell cardiomyocytes (average cardiomyocytes in each sample = 1,586; Figures 4A, B), with almost absence of *SPEG* expression in other single cell types, including adipocytes, fibroblast cells, immune cells, lymphatic endothelial cells, macrophages, pericytes, and vascular endothelia cells. Of note, VSMC was an exception, which was found to express *SPEG* but the average number of VSMC in each sample was only ∼10 (Figure 4C).

To determine sex-biased differences among COVID-19 and healthy samples by sex, we conducted sample level analysis by separating samples into four groups, including COVID-19 females, COVID-19 males, healthy females, and healthy males, and evaluated cell counts and cell percentage of each single cell type across these four groups by individual. We revealed that both COVID-19 females and males had lower counts of cardiomyocytes compared to healthy individuals, but only between COVID-19 males and healthy females, the number of cardiomyocyte counts was significantly different (Figure 4C). Further comparisons of cell percentage for each single cell type across these four groups revealed that both COVID-19 females and males show lower cardiomyocyte percentage compared to healthy females (adjusted *p* values <0.05; Figure 4D). Surprisingly, we found the percentages of vascular endothelial cells are significantly higher in both COVID-19 females and males than both healthy females and males (all adjusted *p* values <0.05; Figure 4D). However, no differences for the percent of vascular endothelial cells were observed between females and males of COVID-19 or healthy individuals. We further dissected the cell count and percentage of each single cell type expressing *SPEG* [log2 (normalized expression +1) > 0] in the data set and observed no significant variations for the two parameters in cardiomyocytes and other single cell types (Figures 4E, F). Furthermore, we determined the upregulation of *SPEG* expression in cardiomyocytes by comparing these four groups (Figure 4G). We confirmed that the increased fold change was higher in female COVID-19 patients compared to both healthy females and males (multiple adjusted *p* values <0.05). When compared COVID-19 females with COVID-19 males, the *SPEG* expression is higher in females (fold change = 1.55), although the multiple adjusted *p*-value is not significant (*p* = 0.3).

Furthermore, we performed differential gene expression analysis for *SPEG* at single cell level by collecting all cells of a specific cell type from a group of individuals across four patient groups, including COVID-19 females, COVID-19 males, healthy females, and healthy males. It turned out that the strategy is more robust, as more significant upregulation of *SPEG* in cardiomyocyte was observed in COVID females (0.29 percent of cardiomyocytes expression *SPEG*) than in COVID-19 males (0.22 percent of cardiomyocytes expression *SPEG*) (differential expression *p*-value = 5.0e-18 and fold change = 1.2; see Figure 4H; Supplementary Figure S1). Detailed evaluation of *SPEG* expression in each individual sample (Supplementary Figure S1) revealed that three out of six COVID-19 females shown higher upregulation of *SPEG* in cardiomyocytes, with another 2 COVID-19 females contained <100 cardiomyocytes. Meanwhile, among 12 COVID-19 males, there are two COVID-19 males with no cardiomyocytes detected in the single cell data, seven COVID-19 males demonstrated lower expression of *SPEG*, and two male COVID-19 individuals displayed higher *SPEG* expression comparable to COVID-19 females. The lack of enough cardiomyocytes in the two female COVID-19 individuals and the higher expression of *SPEG* in two COVID-19 males are the reason why the insignificant difference at sample level was observed between COVID-19 females and males in terms of *SPEG* expression. In addition, COVID-19 females but not COVID-19 males demonstrated significantly higher expression of *SPEG* compared to these healthy females and males (Figure 4H; all *p* values <1e-36). Individual level single cell expression of *SPEG* in cardiomyocyte confirmed that majority of healthy females and males demonstrated lower expression of *SPEG* (Supplementary Figure S1). In terms of VSMCs, although aggregating all single cells by patient group increased the differences of *SPEG* expression observed between COVID-19 males and either of healthy males and healthy females (both *p* values = 2.2e-15), there are few VSMC cells expressing *SPEG* among all these individual samples and no significant difference in terms of *SPEG* expression in VSMCs found between females and males of COVID-19. The significant difference for VSMCs may be driven by two COVID-19 males with more VSMCs showing relatively higher expression of *SPEG* compared to all other healthy individuals. Thus, the significant up-regulation of VSMCs in COVID-19 males is not conclusive.

Finally, we evaluated *SPEG* expression in a bulk RNAseq data of cardiac cells upon SARS-CoV-2 infection. The bulk RNAseq dataset consists of multiple cardiac cells, including human induced pluripotent stem cell-derived cardiomyocytes, cardiac fibroblasts, and endothelial cells. We only observed that the significant up-regulation of *SPEG* upon SARS-CoV-2 infection with high multiplicity of infection equal to 0.1 in cardiomyocytes (Supplementary Figure S2).

To this end, both in single cell and bulk RNAseq analysis, *SPEG* is specifically expressed in heart cardiomyocytes, and upon SARS-CoV-2 infection, *SPEG* is up-regulated in cardiomyocytes, with only statistically significant upregulation of *SPEG* in COVID-19 females but not in COVID-19 males compared to either female or male healthy controls at both sample level and single cell level analyses.

## Discussion

Growing evidence suggests that hypertension is the frequently observed comorbidity with severe COVID-19 (Wang et al., 2020; Yang et al., 2020; Gallo et al., 2022). Through integrative screening of genetic factors associated with both hypertension and severe COVID-19, we revealed that the cis-eQTL rs12474050 of *SPEG* is a potential host factor predisposing to higher DBP and severe COVID-19 in women. The risk allele rs12474050*T correlates with lower expression of *SPEG* in multiple tissues, including muscle skeletal, heart atrial appendage, and heart left ventricle. Among 49 GTEx tissues, higher expression of *SPEG* was detected in muscle skeletal, heart atrial appendage, and heart left ventricle of females compared to males, potentially suggesting *SPEG* expression is under sex-biased regulation. Coincidently, Fagerberg et al. and Singh et al. demonstrated *SPEG* was highest decoded in endometrium (Fagerberg et al., 2014; Singh et al., 2020). The close relationship between endometrium and cardiomyocyte was presented by Fan et al. that the endometrium-derived stem cells could repair myocardial ischemia injury (Fan et al., 2021). In our study, we further confirmed that higher *SPEG* expression in breast mammary tissue between females and males. Such observations rationalize our hypothesis that *SPEG* is highly sex-biased with severe COVID-19 in women. Compared to males, females tended to have higher risk for heart related diseases, such as atrial fibrillation (Chugh et al., 2013; Emdin et al., 2016). Females preferred to suffer from long COVID than males since immune system of females is more sensitive upon viral infection (Ganesh et al., 2022). Our analyses implicated the interplay among sex, hypertension, cardiovascular diseases, and severe COVID-19. This might be due to the important functions of *SPEG* in heart, especially for its potential protective roles in cardiomyocytes in COVID-19. Notably, the post-acute cardiovascular manifestations of COVID-19 were widely reported (Xie et al., 2022) and the putative mechanisms interestingly involved fibrosis and scarring of cardiac tissue caused by activated TGF-β signaling (Mustroph et al., 2021). Coincidently, we revealed that upon SARS-CoV-2 infection the expression of *SPEG* was upregulated in cardiomyocytes, implicating the potential damage in heart in COVID-19. As SARS-CoV-2 infection could damage the cardiac tissue both directly and indirectly (Topol, 2020; Saeed et al., 2021), the upregulation of *SPEG*, partially increased in higher magnitude in female cardiomyocytes, would be a potentially compensatory response and self-cardioprotective action due to the infection of SARS-CoV-2 in heart. Taken together, the expression of *SPEG* is influenced by sex and SARS-CoV-2 infection, and *SPEG* is a critical host factor involved in severe COVID-19 in women.

Previous studies advocated that *SPEG* played pivotal roles in the development, maintenance, and function of cardiac and skeletal muscles (Campbell et al., 2021; Luo et al., 2021). The *SPEG* gene encoded SPEG belongs to the Unc89 subfamily, myosin light chain kinase (MLCK) protein family (Geisler et al., 2007). The Unc89 subfamily members could induce phosphorylation of junctophilin 2 (JPH2), ryanodine receptor (RyR2), sarcoplasmic/endoplasmic reticulum Ca^2+^ ATPase 2a (SERCA2a), and α-tropomyosin (TPM1) in cardiac muscle (Campbell et al., 2021). As a result, *via* the triggering of phosphorylation by SPEG, the JPH2, RyR2, SERCA2a, and TPM1 were tightly involved in excitation–contraction (E-C) coupling (Bers, 2008; Landstrom et al., 2017). The E-C coupling is a key physiological process of conversion of electrical stimuli into a mechanical action in skeletal muscle contraction and is a core phenotype as one of the biological functions of SPEG. *SPEG* mutations have been found in patients with centronuclear myopathy which is an inherited neuromuscular disorder characterized by clinical features of a congenital myopathy and centrally placed nuclei on muscle biopsy (Agrawal et al., 2014). In addition, the lower expression of *SPEG* was observed in human end-stage HF (Quick et al., 2017). Further *in vivo* studies suggests that SPEG is essential for proper myocyte formation and maturation, and for cardiac development and function (Ono et al., 2005). Consequently, Quick et al. reported that specific tamoxifen-inducible acute down-regulation of *SPEG* in cardiomyocytes of over 8 weeks mice resulted in disruption of transverse tubule integrity, impaired calcium handling, altered E-C coupling, and HF (Quick et al., 2017). Based on the abundant pieces of information for critical role of *SPEG* in cardiovascular system, we confirmed the enhanced expression of *SPEG* in female but not male would be protective against severe COVID-19.

Our integrative genetic screening of SNPs associated with both hypertension and severe COVID-19 revealed that the cis-eQTL rs12474050 of *SPEG* was potentially associated with severe COVID-19 in women. Both single cell RNAseq and bulk RNAseq analyses confirmed the upregulation of *SPEG* in cardiac cell type cardiomyocytes upon SARS-CoV-2 infection. Furthermore, *SPEG* expression was higher in normal heart tissues of females than that of males. In addition, *SPEG* was upregulated in cardiomyocytes of female COVID-19 patients compared to both healthy females and males. Our finding suggested that *SPEG* plays a protective role in heart damage of female COVID-19 patients. This has even broad implication regarding to the substantially increased risk of heart disease in COVID-19 survivors according to two recent large-scale studies, including a 2021 study based on 13,638 health records (Mainous et al., 2021) from Florida and a 2022 study considering 153,760 COVID-19 survivors and thousands of controls (Xie et al., 2022). COVID-19 might induce cardiac injury through systemic inflammation and ischemic pathways, also including stress cardiomyopathy, acute and fulminant myocarditis (Abbasi, 2021). In our study, the percentage of vascular endothelial cells in heart tissue from COVID-19 patients of both sex is dramatically increased, which is consistent with previous report that vascular endothelia dysfunction is commonly associated with COVID-19 induced heart failure (Otifi and Adiga, 2022). Nevertheless, female-gender differences in cardiovascular diseases from SARS-CoV-2 were not fully understood. Some reports demonstrated these may be due to COVID-19 induced microvascular coagulopathy and worsening consequent thrombocytopenia (Muir et al., 2021; Bechmann et al., 2022) thereafter to cause heart damage. Additionally, stress cardiomyopathy was frequently found in COVID-19 female patients (Magadum and Kishore, 2020). Regarding to the crucial function of *SPEG* in heart-left ventricular E-C coupling, we deduce that *SPEG* is involved in severe COVID-19 or long COVID induced heart damage particular in woman by providing protective roles. Further investigations using cardiomyocytes with *SPEG* knockout, the tamoxifen treatment mouse model, or an inducible heart-specific *SPEG* knockout/overexpression mouse model will be warranted in order to systematically delineate the exact mechanisms for genetic functional variants in female severe COVID-19 patients.

Our study has limitations. One limitation is that we are not able to infer the potential causal relationship between hypertension and sex-biased severe COVID-19 in women. Performing colocalization analysis to infer causal relationship between hypertension and severe COVID-19 requires to have GWAS summary statistics from both hypertension and sex-stratified severe COVID-19 GWASs (Foley et al., 2021). Unfortunately, the hypertension GWAS summary statistics is not freely available, and thus we are not able to conduct the analysis in current study. However, we found that rs12474050 is an independent genome-wide significant SNPs in the hypertension GWAS, and in the sex-stratified severe COVID-19 GWAS the SNP is also one of the top signals associated with severe COVID-19 in female patients (Figure 1B). Therefore, we trust that rs12474050 is a potential SNP associated with both hypertension and COVID-19 in females. Nevertheless, given the small sample size of severe female COVID-19 patients in the current severe COVID-19 GWAS, further replication of the severe COVID-19 association in women needs to be conducted first before evaluating the causal relationship between hypertension and severe COVID-19 in females, as well as between hypertension or severe COVID-19 and *SPEG* gene expression. In addition, other important hypertension signals may be excluded in our analysis. A more comprehensive analysis would be to search for all hypertension signals from all published GWAS studies in the sex-stratified severe COVID-19 GWASs and subsequently perform sex-biased analysis for these curated hypertension SNPs in COVID-19. Alternatively, it is also feasible to carry out genome-wide cross-trait meta-analysis between sex-biased COVID-19 GWASs and hypertension GWAS according to cai et al. (Cai et al., 2022). The other limitation is that rs12474050 is also a missense variant of *SPEGNB*, which is also named as *SPEG* neighbor. However, the expression data of *SPEGNB* is not included in the GTEx portal due to it is expression level is extremely low across 49 tissues. Further evaluation of its expression in the human protein expression atlas (Uhlén et al., 2015) confirmed that it is only lowly and specifically expressed in the tongue and muscle skeletal tissue, among which the TPM of *SPEGNB* is less than 3. In addition, the function of *SPEGNB* is still undetermined. The potential involvement of *SPEGNB* in both hypertension and severe COVID-19 as well as it is relationship with *SPEG* warrants for further study. The third limitation is about the exact reason of the low percentage of cardiomyocytes expressing *SPEG* in both the heart samples from COVID-19 patients and healthy control is not clear. Although potential sub-clusters of cardiomyocytes may specifically express *SPEG*, we could not find evidences to support this. In details, in the heart single cell data set, we find the cardiomyocytes that express *SPEG* tend to be evenly distributed in the cardiomyocyte cluster (Figure 4B), and all cardiomyocytes are clustered tightly together in the UMAP, suggesting no potential sub-clusters exist among cardiomyocytes. The above conclusion is further confirmed after re-analyzing the single cardiac cell data of ‘Cardiac Differentiation’ published by Kathiriya et al. (Kathiriya et al., 2021) *via* UCSC Cell Browser. Kathiriya et al. studied the knockout of an important cardiac cell development transcription factor *TBX5* by comparing single cell expression data of wild type of human induced pluripotent stem cell (iPSC) lines, Crispr-cas9-exposed control, with two Crispr-cas9-enginered *TBX5* knockout iPSC lines, including one single copy knockout and two-copy knockout of *TBX5* iPSC lines. They generated single cell data sets of iPSC lines developed to cardiomyocytes in a range of time points, including 6 days, 11 days, and 23 days. By performing trajectory inference analysis on the single cell data (Supplementary Figure S3), we confirm that *SPEG* tend to be highly expressed at the time point 11 days, with lower expression of *SPEG* detected at 3 days and no expression of *SPEG* observed at 23 days. We also find that *SPEG* is evenly expressed among cardiomyocytes at the time point 11 days and no obvious sub-clusters specifically expressing *SPEG*. In conclusion, it would be difficult to determine cardiomyocyte sub-clusters in the current heart single cell data sets, mainly due to the expression characteristics of *SPEG* during cardiomyocyte development. Future research is warranted to address the above problem.

## Data Availability

Publicly available datasets were analyzed in this study. Used data can be found in the text.

## Supplementary material

Supplementary Figure S1 Violin plots and Uniform Manifold Approximation and Projection (UMAP) plots for SPEG expression at single sample level. **(A,B)** illustrate SPEG expression in each individual sample for two cell types, including cardiomyocyte and vascular smooth muscle cell (VSMC), respectively. These individuals are categorized into four patient groups, including COVID-19 females, COVID-19 males, healthy females, and healthy males, which are indicated by different color bars at the bottom of these violin plots. SPEG expression is further evaluated at single simple level using UMAP plots for the four groups, including COVID-19 females **(C)**, COVID-19 males **(D)**, healthy females **(E)** and healthy males **(F)**. Note: R package Seurat (Hao et al., 2021) was used to perform single cell analysis at single sample level.

Supplementary Figure S2 Bulk RNAseq analysis of multiple cardiac cell lines, including human induced pluripotent stem cell-derived cardiomyocytes, cardiac fibroblasts, and endothelial cells, reveal that SPEG expression is up-regulated in cardiomyocytes upon SARS-CoV-2 infection. Among multiple cardiac cell lines, including human induced pluripotent stem cell (iPSC), iPSC-derived cardiomyocytes (Cardiomyocytes), Cardiac fibroblasts, and Endothelial cells, *SPEG* expression is increased in cardiomyocytes upon SARS-CoV-2 infection, compared to its corresponding mock controls. The data set was published by Perez-Bermejo et al., cells were treated with either vehicle or SARS-CoV-2 virus for 48 hours at a MOI of either 0.0006 (low), 0.01 (middle), or 0.1 (high) (Perez-Bermejo et al., 2021). Note: Pairwise differential expression analysis were performed with the Student’s t-test, with the unadjusted statistical significance represented by * (*p* ≤ 0.05), ** (*p* ≤ 0.01), *** (*p* ≤ 0.001), and **** (*p* ≤ 0.0001); MOI: multiplicity of infection. The multiple adjusted p value threshold is set as *p* < 0.008 (0.05 / 6), thus, only the comparison of *SPEG* expression between high MOI SASR-CoV-2 infection and its corresponding mock control passes the multiple adjusted p value threshold.

Supplementary Figure S3 Single cell trajectory inference analysis for SPEG expression in cardiac cells using UCSC Cell Browser. Three panels, including **(A–C)**, illustrating the UMAP for all single cells (upper part), UMAP of SPEG expressed cells (middle part) , and pseudotime analyses for SPEG expressed cells and all cells, with cells are colored by SPEG expression level or by different sample source, respectively (see lower two consecutive parts). The single cell data were derived from the following 4 groups: wild type of human induced pluripotent stem cell (iPSC) lines, Crispr-cas9-exposed control lines, and two Crispr-cas9-enginered TBX5 knockout iPSC lines, including one single copy knockout and two-copy knockout of TBX5 iPSC lines, for 3 time points of 6 days, 11 days, and 23 days. The data set was published by Kathiriya et al. (Kathiriya et al., 2021) and is named as “Cardiac Differentiation” at UCSC Cell Browser. Note: on day 23, SPEG expression is not detectable.
