# Supplementary figures and images for: Genetic screening for hypertension and COVID-19 reveals functional variation of SPEG potentially associated with severe COVID-19 in women
Source: Front Genet. 2023 Jan 4;13:1041470. doi: 10.3389/fgene.2022.1041470 (PMC9846087; doi:10.3389/fgene.2022.1041470)

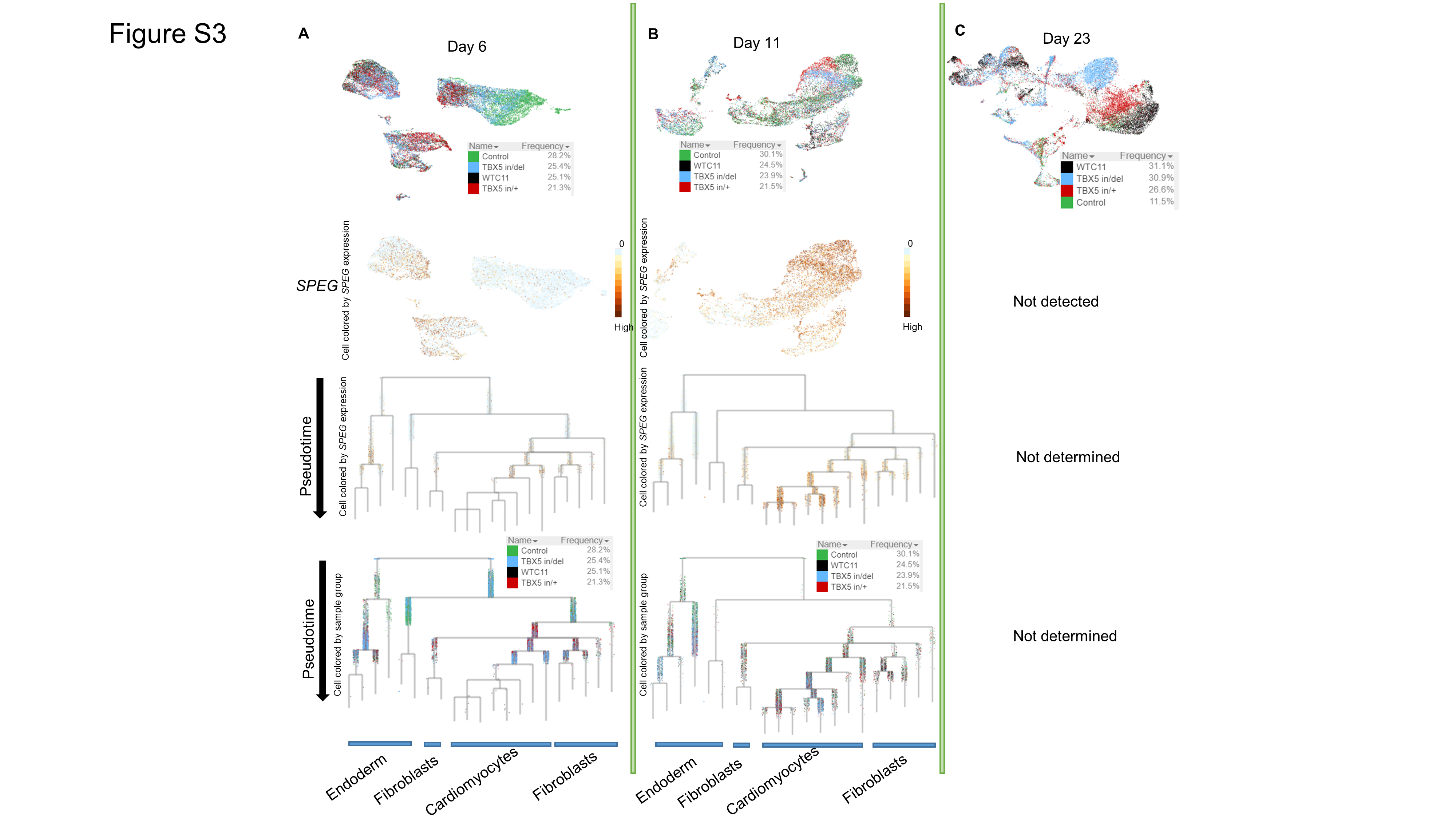

Supplement: Supplementary file 1 [file Image6.tif]

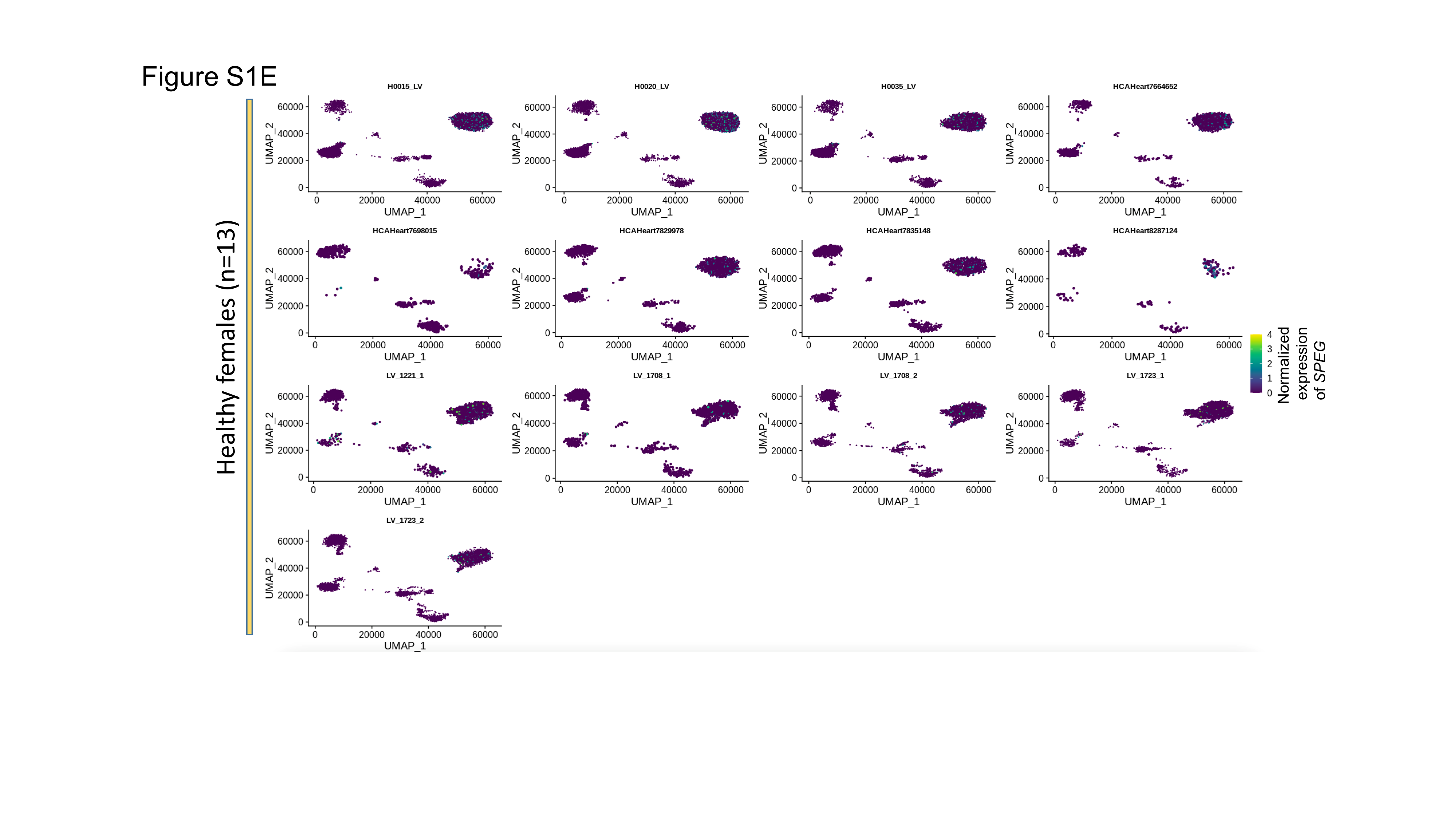

Supplement: Supplementary file 2 [file Image3.tif]

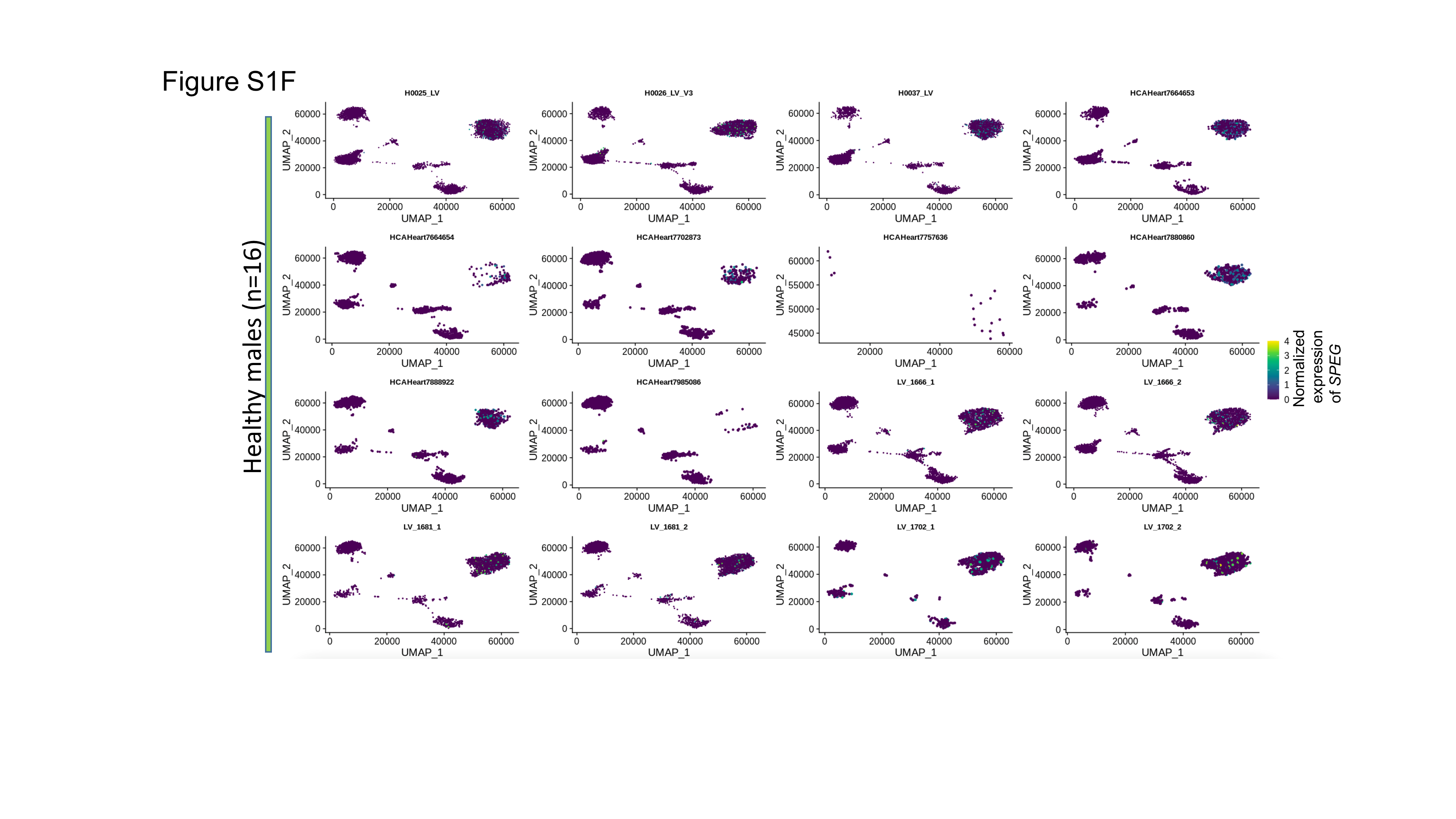

Supplement: Supplementary file 3 [file Image4.tif]

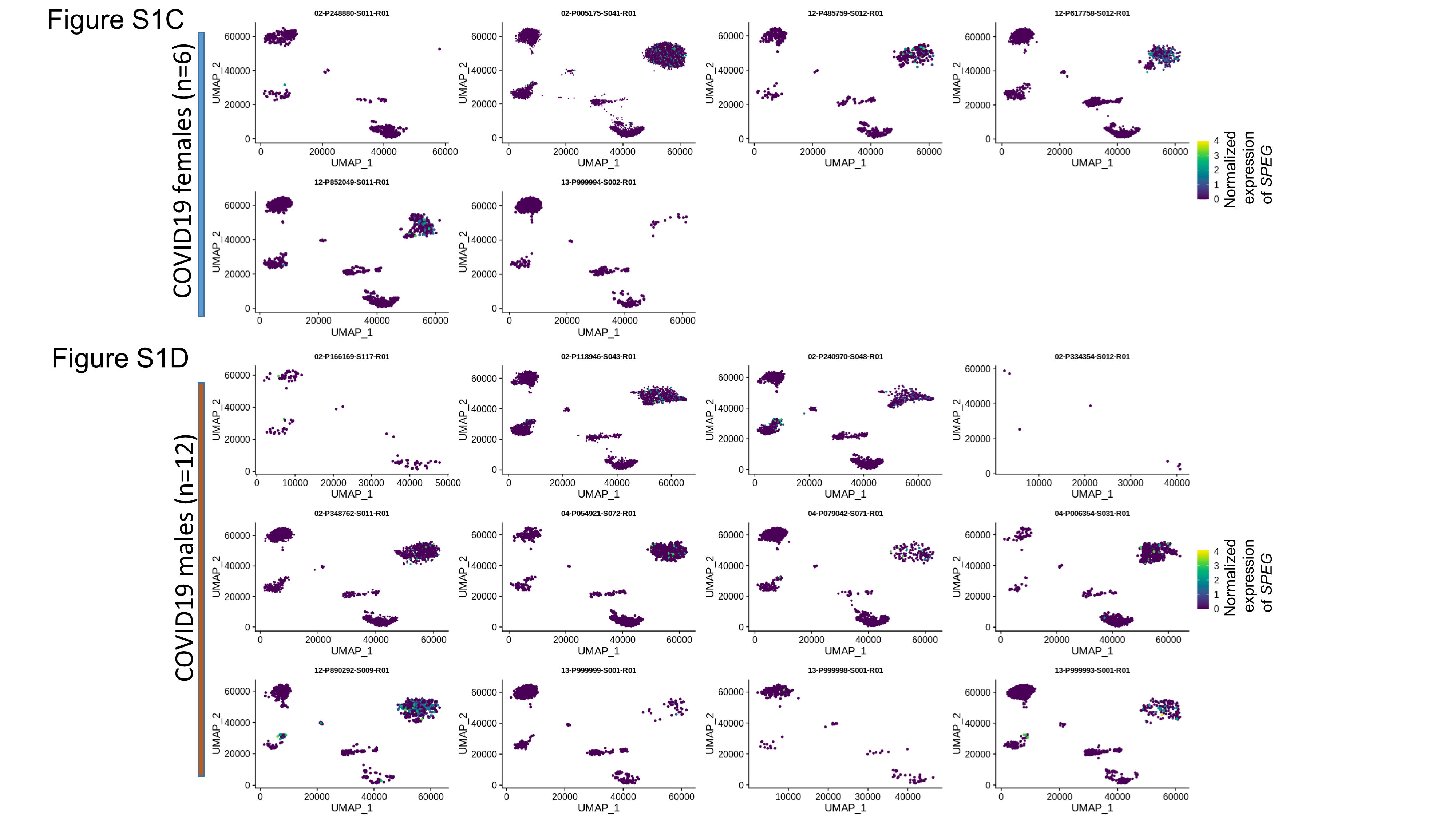

Supplement: Supplementary file 4 [file Image2.tif]

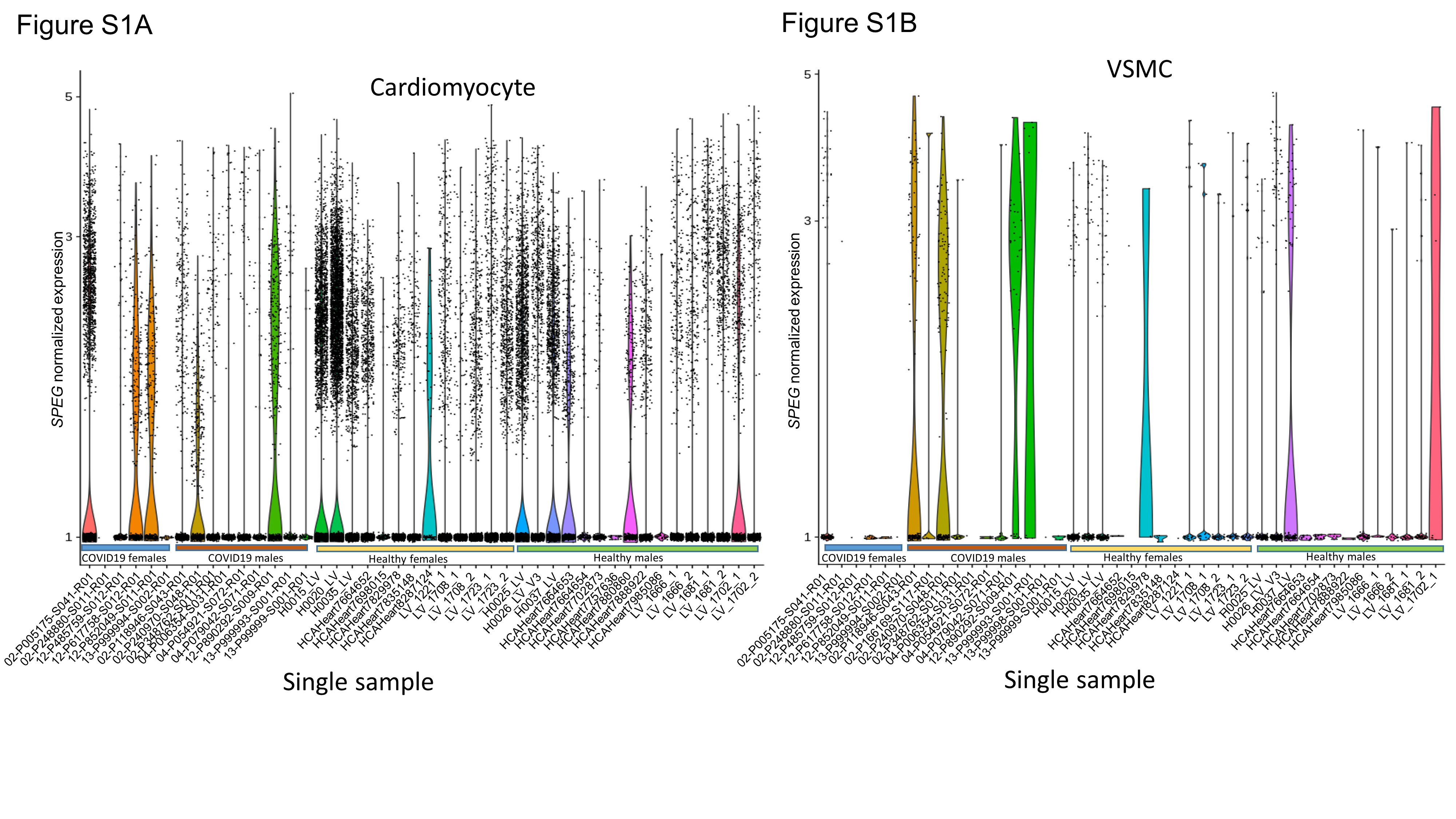

Supplement: Supplementary file 5 [file Image1.tif]

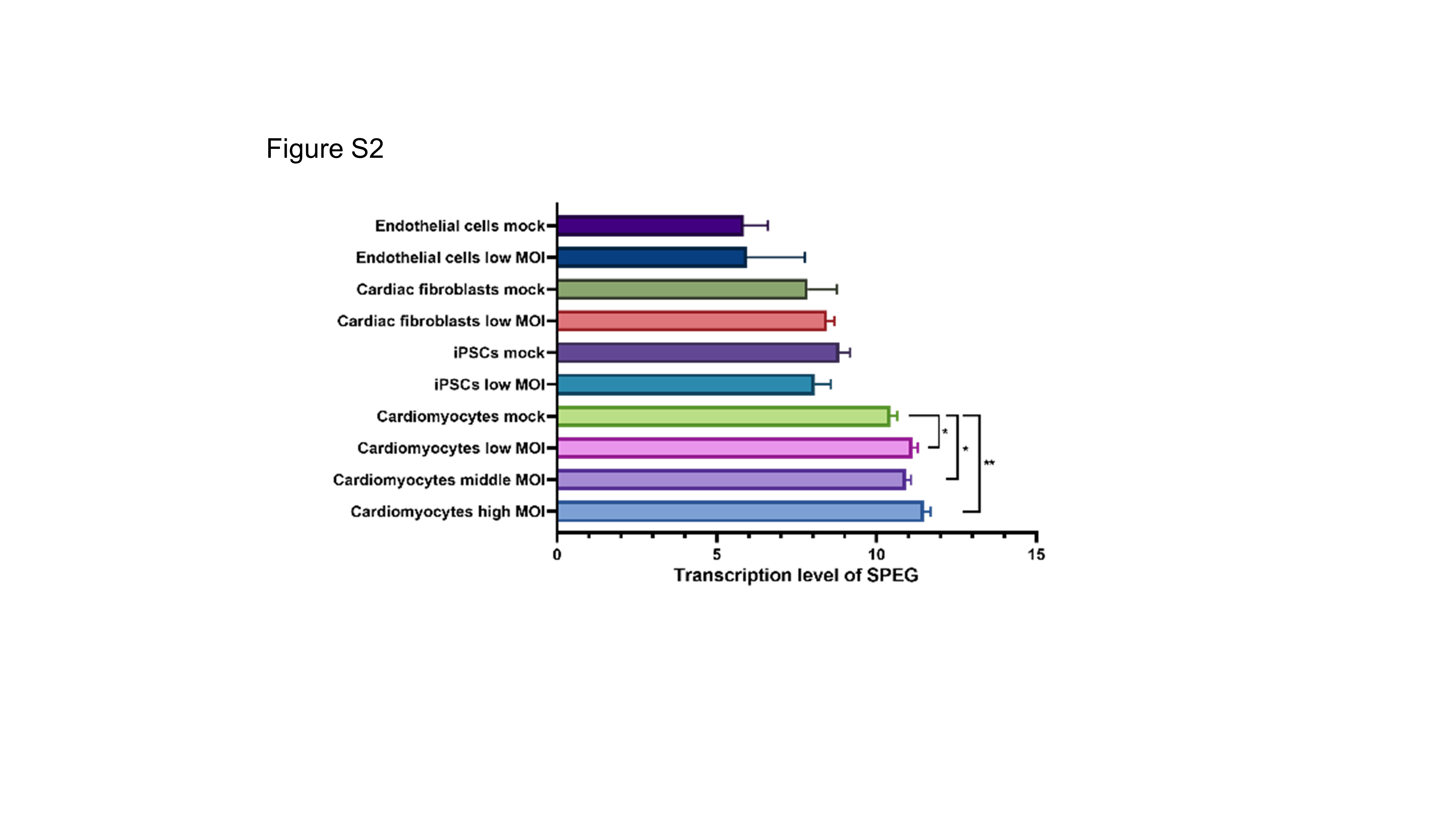

Supplement: Supplementary file 6 [file Image5.tif]
