# Supplementary material for: Genetic screening for hypertension and COVID-19 reveals functional variation of SPEG potentially associated with severe COVID-19 in women
Source: Front Genet. 2023 Jan 4;13:1041470. doi: 10.3389/fgene.2022.1041470 (PMC9846087; doi:10.3389/fgene.2022.1041470)
Supplement: Supplementary file 7 [file DataSheet1.docx]

**Supplementary Materials**

**Materials and Methods**

**Thirteen COVID-19 single cell data sets from UCSC Cell Browser**

The 13 COVID-19 related single cell data sets available from UCSC Cell Browser are ‘SARS-CoV-2 Target Cells in Human Airway Epithelium’ [1], ‘SARS-CoV-2 and Cigarette Smoke’ [2], ‘COVID-19 PBMC’ [3], ‘COVID-19 and Influenza Immunophenotyping’ [4], ‘Monocyte-associated Cytokine Storm in Severe COVID-19’ [5], ‘Immune cells in Critical COVID-19 Cases’ [6], ‘COVID-19 Airways’ [7], ‘Bronchoalveolar Immune Cells in COVID-19’ [8], ‘Cellular Targets of SARS-CoV-2’ [9, 10], ‘COVID-19 Immunological Response’ [11], ‘Immune Cells in Severe COVID-19’ [12], ‘COVID-19 in Hypertensive Patients’ [13], and ‘Peripheral Immune in COVID-19 Infection’ [14].

**Differential expression analysis for *SPEG* in the heart single cell data set using R package Seurat**

The single cell expression read count matrix of heart tissues downloaded from UCSC Cell Browser was read into R and created as a Seurat object using the function ‘CreateSeuratObject’ with default setting of minimum 3 cells in a single sample and minimum 200 genes in a cell. Subsequently, the sample and single cell meta data were added into the above Seurat object via the function ‘AddMetaData’ by matching the single cell ids between the cell expression read count matrix and the meta file. Similarly, we incorporated the UMAP coordinates of these single cells by looking up cell ids between the above Seurat object and UMAP file using the function ‘CreateDimReducObject’. Then we followed the standard protocol of Seurat to analyze these single cell data by performing normalization again with default setting of ‘LogNormalize’ via the function ‘NormalizeData’. We used the function ‘Vlnplot’ to visualize *SPEG* expression for each single cell type among the four groups, including COVID-19 females, COVID-19 males, healthy females, and healthy males. In addition, we also used the same function to evaluate *SPEG* expression in a specific single cell type by sample, in which way we can determine which samples in each of the four patient groups show similar or different distribution of *SPEG* expression. For all samples, we applied the function ‘FeaturePlot’ to demonstrate all single cells of a specific single cell type that express *SPEG* via UMAP, which is a good method to detect samples with few total numbers of cells or few cells of a specific cell type among the four patient groups. The function ‘subset’ and ‘FindMarkers’ were specifically used to extract single cell expression data of *SPEG* among a specific cell type and paired patient groups by sexes and COVID-19 status, and to perform differential expression analysis using the ‘wilcox’ method (Wilcoxon Rank Sum test) for *SPEG* with default setting, with the adjusted p values provided by the function were used. Additionally, the average log2(fold change) of gene expression and the percentage of cells expressing a specific gene in two compared patient groups were generated by the function ‘FindMarkers’.

**Data availability**

Candidate hypertension cis-eQTLs (n=172) from the hypertension GWAS (read the sheet “ST9” in the excel file):

<https://static-content.springer.com/esm/art%3A10.1038%2Fs41588-018-0205-x/MediaObjects/41588_2018_205_MOESM3_ESM.xlsx>

GRASP database:

<https://grasp.nhlbi.nih.gov/Covid19GWASResults.aspx>

Summary statistics of severe COVID-19 GWAS of females downloaded from GRASP:

<https://grasp.nhlbi.nih.gov/downloads/COVID19GWAS/02242021/UKBB_severe_EURpstv_F_022421.txt.gz>

Summary statistics of severe COVID-19 GWAS of males downloaded from GRASP:

<https://grasp.nhlbi.nih.gov/downloads/COVID19GWAS/02242021/UKBB_severe_EURpstv_M_022421.txt.gz>

GTEx bulk RNAseq TPM matrix data derived from 49 tissues:

<https://storage.googleapis.com/gtex_analysis_v8/rna_seq_data/GTEx_Analysis_2017-06-05_v8_RNASeQCv1.1.9_gene_tpm.gct.gz>

Sex information for these 49 tissues from GTEx:

<https://storage.googleapis.com/gtex_analysis_v8/annotations/GTEx_Analysis_v8_Annotations_SubjectPhenotypesDS.txt>

GTEx tissue information covering sample IDs and tissue IDs for these 49 tissues:

<https://storage.googleapis.com/gtex_analysis_v8/annotations/GTEx_Analysis_v8_Annotations_SampleAttributesDS.txt>

Single cell visualization tool from GTEx Portal:

<https://www.gtexportal.org/home/multiGeneSingleCellQueryPage>

Heart tissue single cell data set provided by UCSC Cell Browser via these links:

Normalized expression matrix:

<https://cells.ucsc.edu/covid19-cellular-targets/heart/all/exprMatrix.tsv.gz>

Heart sample and different single cell type meta data:

<https://cells.ucsc.edu/covid19-cellular-targets/heart/all/meta.tsv>

Information of Uniform Manifold Approximation and Projection [UMAP] for all single cells:

<https://cells.ucsc.edu/covid19-cellular-targets/heart/all/UMAP.coords.tsv.gz>.

SARS-CoV-2 infection bulk RNAseq dataset GSE156754 downloaded from GEO:

<https://www.ncbi.nlm.nih.gov/geo/download/?acc=GSE156754&format=file>

**References**

1. Ravindra, N.G., et al., *Single-cell longitudinal analysis of SARS-CoV-2 infection in human airway epithelium identifies target cells, alterations in gene expression, and cell state changes.* PLoS biology, 2021. **19**(3): p. e3001143.

2. Purkayastha, A., et al., *Direct exposure to SARS-CoV-2 and cigarette smoke increases infection severity and alters the stem cell-derived airway repair response.* Cell Stem Cell, 2020. **27**(6): p. 869-875.

3. Stephenson, E., et al., *Single-cell multi-omics analysis of the immune response in COVID-19.* Nature Medicine, 2021. **27**(5): p. 904-916.

4. Lee, J.K., et al., *Immunophenotyping of COVID-19 and influenza highlights the role of type I interferons in development of severe COVID-19.* Science Immunology, 2020. **5**(49): p. eabd1554.

5. Guo, C., et al., *Single-cell analysis of two severe COVID-19 patients reveals a monocyte-associated and tocilizumab-responding cytokine storm.* Nature Communications, 2020. **11**(1): p. 3924.

6. Ren, X., et al., *COVID-19 immune features revealed by a large-scale single cell transcriptome atlas.* Cell, 2021. **184**(7): p. 1895-1913.

7. Chua, R.L., et al., *COVID-19 severity correlates with airway epithelium–immune cell interactions identified by single-cell analysis.* Nature Biotechnology, 2020. **38**(8): p. 970-979.

8. Liao, M., et al., *Single-cell landscape of bronchoalveolar immune cells in patients with COVID-19.* Nature medicine, 2020. **26**(6): p. 842-844.

9. Melms, J.C., et al., *A molecular single-cell lung atlas of lethal COVID-19.* Nature, 2021. **595**(7865): p. 114-119.

10. Delorey, T.M., et al., *COVID-19 tissue atlases reveal SARS-CoV-2 pathology and cellular targets.* Nature, 2021. **595**(7865): p. 107-113.

11. Zhang, J.Y., et al., *Single-cell landscape of immunological responses in patients with COVID-19.* Nature Immunology, 2020. **21**(9): p. 1107-1118.

12. Liu, C., et al., *Time-resolved systems immunology reveals a late juncture linked to fatal COVID-19.* Cell, 2021. **184**(7): p. 1836-1857.

13. Trump, S., et al., *Hypertension delays viral clearance and exacerbates airway hyperinflammation in patients with COVID-19.* Nature Biotechnology, 2021. **39**(6): p. 705-716.

14. Wilk, A.J., et al., *A single-cell atlas of the peripheral immune response in patients with severe COVID-19.* Nature Medicine, 2020. **26**(7): p. 1070-1076.
